# Supplementary material for: Variable Expression of the Disialoganglioside GD2 in Breast Cancer Molecular Subtypes
Source: Cancers (Basel). 2021 Nov 8;13(21):5577. doi: 10.3390/cancers13215577 (PMC8582848; doi:10.3390/cancers13215577)

Supplementary Materials

Tumor grading

Grading was determined using pre-treatment formalin-fixed, paraffin-embedded (FFPE) breast cancer core biopsies in accordance with the Elston and Ellis criteria [49].

Immunohistochemical staining of ER, PR, HER2, and Ki-67

Immunohistochemistry (IHC) was conducted on FFPE tissue from preoperative core biopsies in accordance with the routine standards in our institute and the manufacturer's instructions, using an automated staining module (BenchMark Ultra IHC/ISH staining module; Ventana Medical Systems, Tucson, Arizona, USA). For assessment of estrogen receptor (ER), progesterone receptor (PR), and Ki-67 IHC status, monoclonal mouse antibodies against ER-alpha (clone 1D5, 1 : 200 dilution; Dako, Glostrup, Denmark), monoclonal mouse antibody against the progesterone receptor (clone pgR636, 1 : 200 dilution; Dako), and monoclonal antibody against Ki-67 (clone MIB-1, 1 : 200 dilution; Dako) were used. The continuous percentage of positively stained tumor cells was stated in the pathology reports; positive staining for ER and PR was time-dependently defined as ≥ 10% and ≥ 1%, respectively [50-53]. The cut-off for proliferation marker Ki-67 was defined as 14% [54].

For HER2 IHC, a polyclonal antibody against HER2 (1 : 200 dilution; Dako) was used, and the HER2 IHC score was documented in the pathology reports as 0, 1+, 2+, or 3+ in accordance with the published guidelines [55]. Tumors with a score of 0 or 1+ were considered as HER2-negative and those with a score of 3+ were defined as HER2-positive. Breast cancer samples with a 2+ staining were analyzed for gene copy numbers of *HER2* using chromogenic in situ hybridization (CISH). The *HER2* gene copy numbers (GCN) and the centromere GCN of the corresponding chromosome 17 were visualized using a kit with two probes of different colors (ZytoDot, 2C SPEC HER2/CEN17; ZytoVision Ltd., Bremerhaven, Germany). A case was regarded as *HER2*-amplified if the *HER2/CEN17* ratio was ≥ 2.2 [56]. Before 2002, patients were retrospectively identified as being HER2-positive or -negative.

The definitions of the subtypes have been reported previously [57]. If the tumor had a HER2 IHC score of 3+ or showed amplification of the *HER2* gene, HER2 status was considered positive (HER2-positive/HER2+ breast cancer) [58]. Patients with negative ER, PR, and HER2 status were defined as having triple-negative breast cancer (TNBC). HER2-negative breast cancers with expression of either ER or PR were further separated into luminal A (-like) tumors (grading of 1 or 2) and luminal B (-like) tumors (grading of 3) [59].

Construction of tissue microarrays

The breast core biopsies were FFPE tissue. Areas containing invasive carcinoma of the breast were marked on a slide stained with hematoxylin–eosin (H&E) by an experienced pathologist. Tissue microarrays (TMAs) were constructed by reembedding cylindric central breast cancer tissue core biopsies (1.0 mm per dot) from several sample donor blocks into a single microarray block at defined coordinates. TMAs with non-neoplastic breast parenchyma from this cohort were created in the same way.

GD2 flow cytometry

Flow cytometry was carried out to investigate possible membranous expression of GD2. Four hormone receptor–positive, HER2-negative breast cancer tissue samples were freshly isolated

and immediately dissociated into single-cell suspension using the gentleMACS tissue dissociator and Tumor Dissociation Kit (both Miltenyi Biotec, Bergisch-Gladbach, Germany) in accordance with the manufacturer's protocol. Cells were filtered twice, washed in phosphate-buffered saline (PBS), and stained with 7-amino-actinomycin D (7-AAD) and antihuman anti-CD45 APC-H7 (clone 2D1), anti-EpCAM FITC (clone EBA-1, all BD Biosciences) and anti-GD2 PE (clone 14G2a, Biolegend) monoclonal antibodies. Cells were determined by FSC-A/SSC-A, doublets were excluded by FSC-H/FSC-A, dead cells were excluded by 7-AAD, and tumor cells were gated by CD45– and EpCAM+. Flow cytometry data were acquired on an FACS LSRFortessa flow cytometer (BD Biosciences, Heidelberg, Germany) and tumor cells were analyzed for GD2 expression using FlowJo software (BD Biosciences). The gating strategy is illustrated in Supplementary Fig. S1.

Controls for GD2 immunohistochemistry

To validate the usability of the GD2 antibody used for FFPE tissue, we compared GD2 immunohistochemistry with flow cytometry results. We a) compared the flow cytometry results of four breast cancer samples (all positive for GD2; see above) with our in-house GD2 IHC protocol. Three samples showed strong GD2 staining in 10%, 40%, and 65-70% of cancer cells, respectively. One sample, however, showed GD2 staining only in the non-neoplastic epithelium close to/intermingled with cancer cells. Furthermore, we b) compared GD2 expression in breast cancer cell lines analyzed by flow cytometry and immunohistochemistry of corresponding cell line FFPE blocks. The breast cancer cell lines MCF7 and HTB-133, which showed both ++ GD2 positivity by flow cytometry, presented with strong staining in 8%, and 40-50%, respectively, using GD2 IHC (Supplementary Fig. S7 1) and 2)). The remaining cell lines showed no or negligible GD2 IHC staining matching with the flow cytometry results (see Supplementary Table S1 and Supplementary Fig. S7 3) and 4)).

Supplementary References

49. Elston, C.W.; Ellis, I.O. Pathological prognostic factors in breast cancer. I. The value of histological grade in breast cancer: experience from a large study with long-term follow-up. Histopathology 1991, 19, 403-410.

50. Goldhirsch, A.; Wood, W.C.; Gelber, R.D.; Coates, A.S.; Thurlimann, B.; Senn, H.J.J.J.C.O. Meeting highlights: updated international expert consensus on the primary therapy of early breast cancer. 2003, 21, doi:10.1200/jco.2003.04.576.

51. Goldhirsch, A.; Glick, J.H.; Gelber, R.D.; Coates, A.S.; Thurlimann, B.; Senn, H.J. Meeting highlights: international expert consensus on the primary therapy of early breast cancer 2005. Annals of oncology : official journal of the European Society for Medical Oncology 2005, 16, 1569-1583, doi:10.1093/annonc/mdi326.

52. Hammond, M.E.; Hayes, D.F.; Dowsett, M.; Allred, D.C.; Hagerty, K.L.; Badve, S.; Fitzgibbons, P.L.; Francis, G.; Goldstein, N.S.; Hayes, M., et al. American Society of Clinical Oncology/College of American Pathologists guideline recommendations for immunohistochemical testing of estrogen and progesterone receptors in breast cancer (unabridged version). Archives of pathology & laboratory medicine 2010, 134, e48-72, doi:10.1043/1543-2165-134.7.e48.

53. Hammond, M.E.; Hayes, D.F.; Wolff, A.C. Clinical Notice for American Society of Clinical Oncology-College of American Pathologists guideline recommendations on ER/PgR and HER2 testing in breast cancer. Journal of clinical oncology : official journal of the American Society of Clinical Oncology 2011, 29, e458, doi:10.1200/jco.2011.35.2245.

54. Goldhirsch, A.; Glick, J.H.; Gelber, R.D.; Coates, A.S.; Senn, H.J. Meeting highlights: International Consensus Panel on the Treatment of Primary Breast Cancer. Seventh International Conference on Adjuvant Therapy of Primary Breast Cancer. Journal of clinical oncology : official journal of the American Society of Clinical Oncology 2001, 19, 3817-3827, doi:10.1200/JCO.2001.19.18.3817.

55. Sauter, G.; Lee, J.; Bartlett, J.M.; Slamon, D.J.; Press, M.F. Guidelines for human epidermal growth factor receptor 2 testing: biologic and methodologic considerations. Journal of clinical oncology : official journal of the American Society of Clinical Oncology 2009, 27, 1323-1333, doi:10.1200/jco.2007.14.8197.

56. Wolff, A.C.; Hammond, M.E.; Schwartz, J.N.; Hagerty, K.L.; Allred, D.C.; Cote, R.J.; Dowsett, M.; Fitzgibbons, P.L.; Hanna, W.M.; Langer, A., et al. American Society of Clinical Oncology/College of American Pathologists guideline recommendations for human epidermal growth factor receptor 2 testing in breast cancer. Archives of pathology & laboratory medicine 2007, 131, 18-43, doi:10.1043/1543-2165(2007)131[18:Asocco]2.0.Co;2.

57. Wunderle, M.; Pretscher, J.; Brucker, S.Y.; Volz, B.; Hartmann, A.; Fiessler, C.; Hein, A.; Häberle, L.; Jud, S.M.; Lux, M.P., et al. Association between breast cancer risk factors and molecular type in postmenopausal patients with hormone receptor-positive early breast cancer. Breast cancer research and treatment 2019, 174, 453-461, doi:10.1007/s10549-018-05115-6.

58. Bethune, G.C.; Veldhuijzen van Zanten, D.; MacIntosh, R.F.; Rayson, D.; Younis, T.; Thompson, K.; Barnes, P.J. Impact of the 2013 American Society of Clinical Oncology/College

of American Pathologists guideline recommendations for human epidermal growth factor receptor 2 (HER2) testing of invasive breast carcinoma: a focus on tumours assessed as 'equivocal' for HER2 gene amplification by fluorescence in-situ hybridization. Histopathology 2015, 67, 880-887, doi:10.1111/his.12723.
59. von Minckwitz, G.; Untch, M.; Blohmer, J.-U.; Costa, S.D.; Eidtmann, H.; Fasching, P.A.; Gerber, B.; Eiermann, W.; Hilfrich, J.; Huober, J., et al. Definition and Impact of Pathologic Complete Response on Prognosis After Neoadjuvant Chemotherapy in Various Intrinsic Breast Cancer Subtypes. Journal of Clinical Oncology 2012, 30, 1796-1804, doi:10.1200/JCO.2011.38.8595.

Supplement Tables

Table S1. Comparison of GD2 expression in breast cancer cell lines analyzed by flow cytometry and GD2 immunohistochemistry of corresponding cell line FFPE blocks. The breast cancer cell lines MCF7 and HTB-133, respectively, presented with matching flow cytometry and IHC results (both positive). The remaining cell lines showed no or negligible GD2 IHC staining, which fitted the flow cytometry results predominantly.

Breast cancer cell line	GD2 flow cytometry	GD2 IHC
MCF7	++	Strong intensity in 8% of tumor cells
HTB-20	-	Intermediate intensity in <1% of tumor cells
HTB-26	-	No staining
HTB-30	+/-	Weak intensity in <0.1% of tumor cells
HTB-132	-	Weak to intermediate intensity in <0.1% of tumor cells
HTB-133	++	Strong intensity in 40-50% of tumor cells

Supplement Figure Legends

Figure S1. The gating strategy in flow cytometry. Cells were determined using FSC-A/SSC-A, doublets were excluded by FSC-H/FSC-A, dead cells were excluded by 7-AAD, and tumor cells were gated by CD45– and EpCAM+.

Figure S2. GD2 is expressed on the surface of tumor cells. Single-cell suspensions of freshly prepared tumor tissues were stained with 7-AAD and anti-CD45, anti-GD2, and anti-EpCAM antibodies. Tumor cells were gated as viable (7-AAD–), CD45–, EpCAM+, and analyzed for GD2 expression (filled histogram) or isotype control (solid line). The histograms show GD2 surface expression on tumor cells from four patients with primary breast cancer.

Figure S3a. Proportions of GD2-positive tumors among breast cancer subtypes using immunohistochemistry (Kruskal–Wallis test, $p < 0.0001$).

Figure S3b. Proportions of GD2-positive tumors among breast cancer subtypes using immunofluorescence (Kruskal–Wallis test, $p < 0.0001$).

Figure S4. Kaplan–Meier curves for GD2 immunohistochemistry (binary) for disease-free survival (DFS) for *(a)* triple-negative breast cancer patients, *(b)* luminal A breast cancer patients, *(c)* luminal B breast cancer patients, and *(d)* HER2-positive breast cancer patients.

Figure S5. Kaplan–Meier curves comparing overall survival (OS) between patients with GD2-positive and GD2-negative tumors among *(a)* triple-negative breast cancer patients, *(b)* luminal A breast cancer patients, *(c)* luminal B breast cancer patients, and *(d)* HER2-positive breast cancer patients.

Figure S6. GD2 immunohistochemistry (IHC) in non-neoplastic breast epithelium (original magnification × 200 in 1 and × 400 in 2). The moderate to strong GD2 positivity in a few noncancerous breast epithelial cells should be noted.

Figure S7. GD2 immunohistochemistry (IHC) of varying breast cancer cell lines. 1) and 2) shows breast cancer cell lines with GD2 positivity: 1) MCF7, and 2) HTB-133 (insets show higher magnification). 3) HTB-132 presented with only weak to intermediate GD2 staining intensity in <0.1% of tumor cells (arrow). 4) HTB-26 did not show any GD2 positivity (original magnification each × 400, insets each × 1500).

Supplement Figures

Figure S1. The gating strategy in flow cytometry. Cells were determined using FSC-A/SSC-A, doublets were excluded by FSC-H/FSC-A, dead cells were excluded by 7-AAD, and tumor cells were gated by CD45– and EpCAM+.

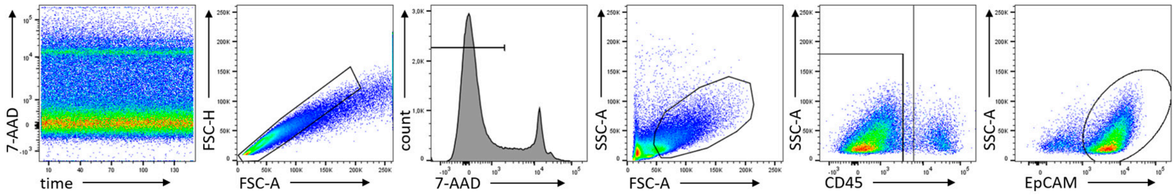

Figure S2. GD2 is expressed on the surface of tumor cells. Single-cell suspensions of freshly prepared tumor tissues were stained with 7-AAD and anti-CD45, anti-GD2, and anti-EpCAM antibodies. Tumor cells were gated as viable (7-AAD–), CD45–, EpCAM+ and analyzed for GD2 expression (filled histogram) or isotype control (solid line). The histograms show GD2 surface expression on tumor cells from four patients with primary breast cancer.

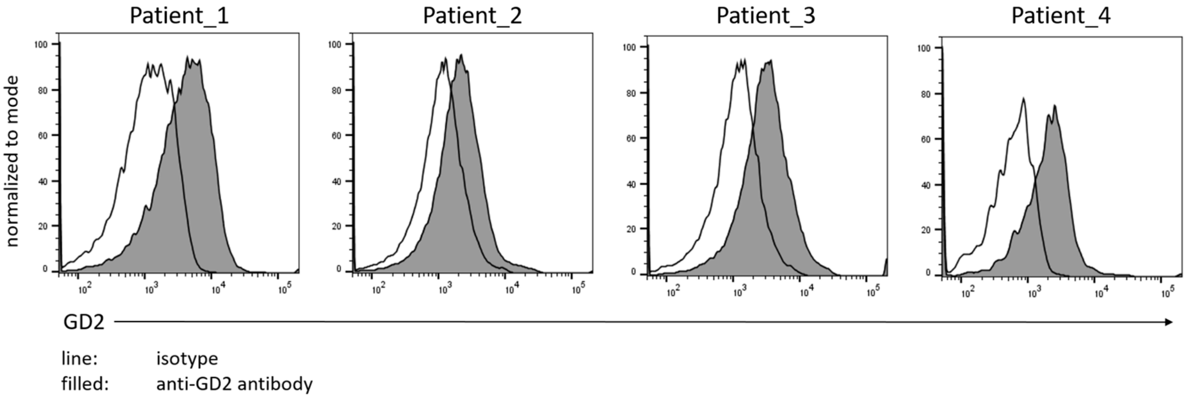

Figure S3a. Proportions of GD2-positive tumors among breast cancer subtypes using immunohistochemistry (Kruskal–Wallis test, $p < 0.0001$).

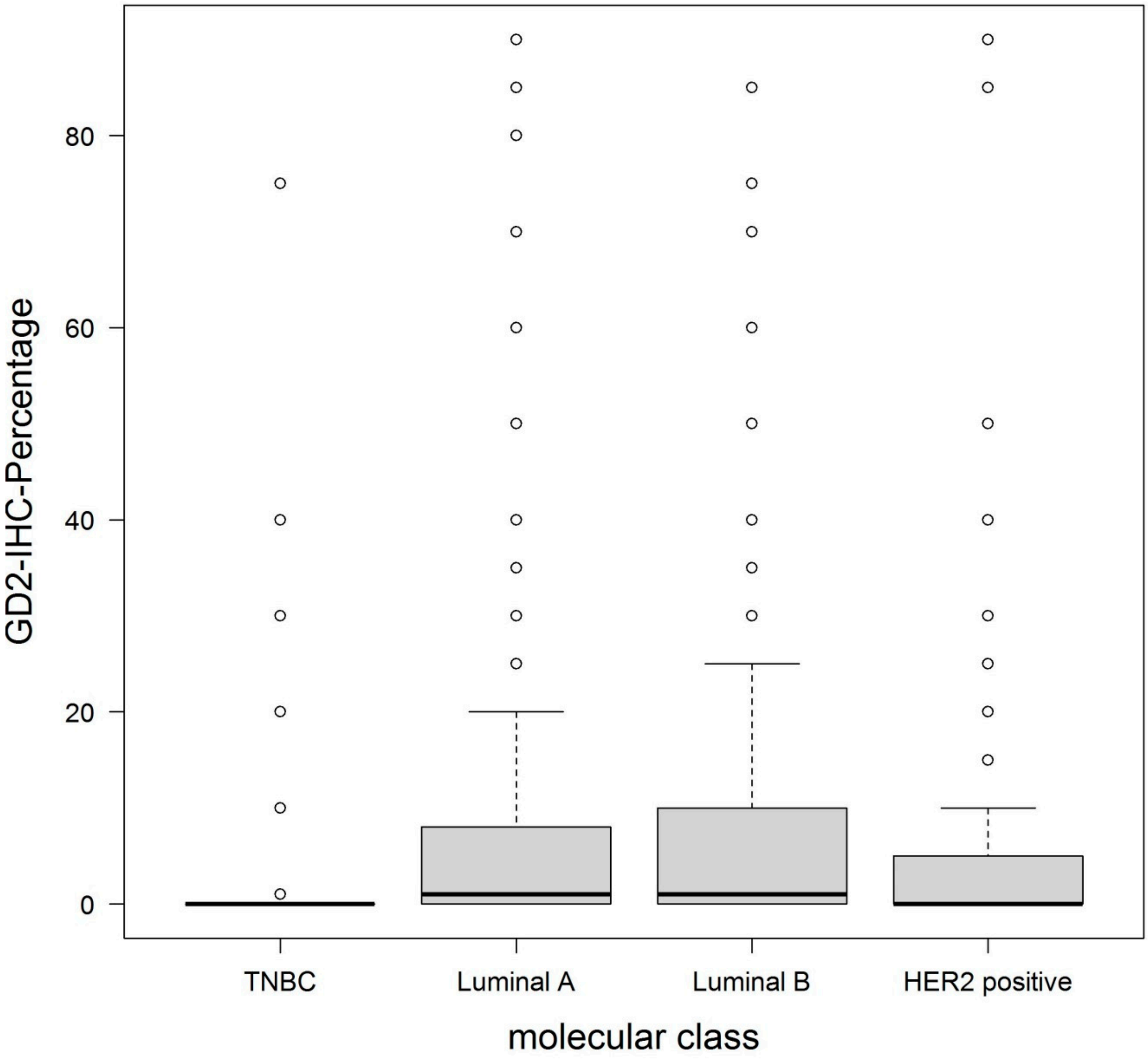

Figure S3b. Proportions of GD2-positive tumors among breast cancer subtypes using immunofluorescence (Kruskal–Wallis test, $p < 0.0001$).

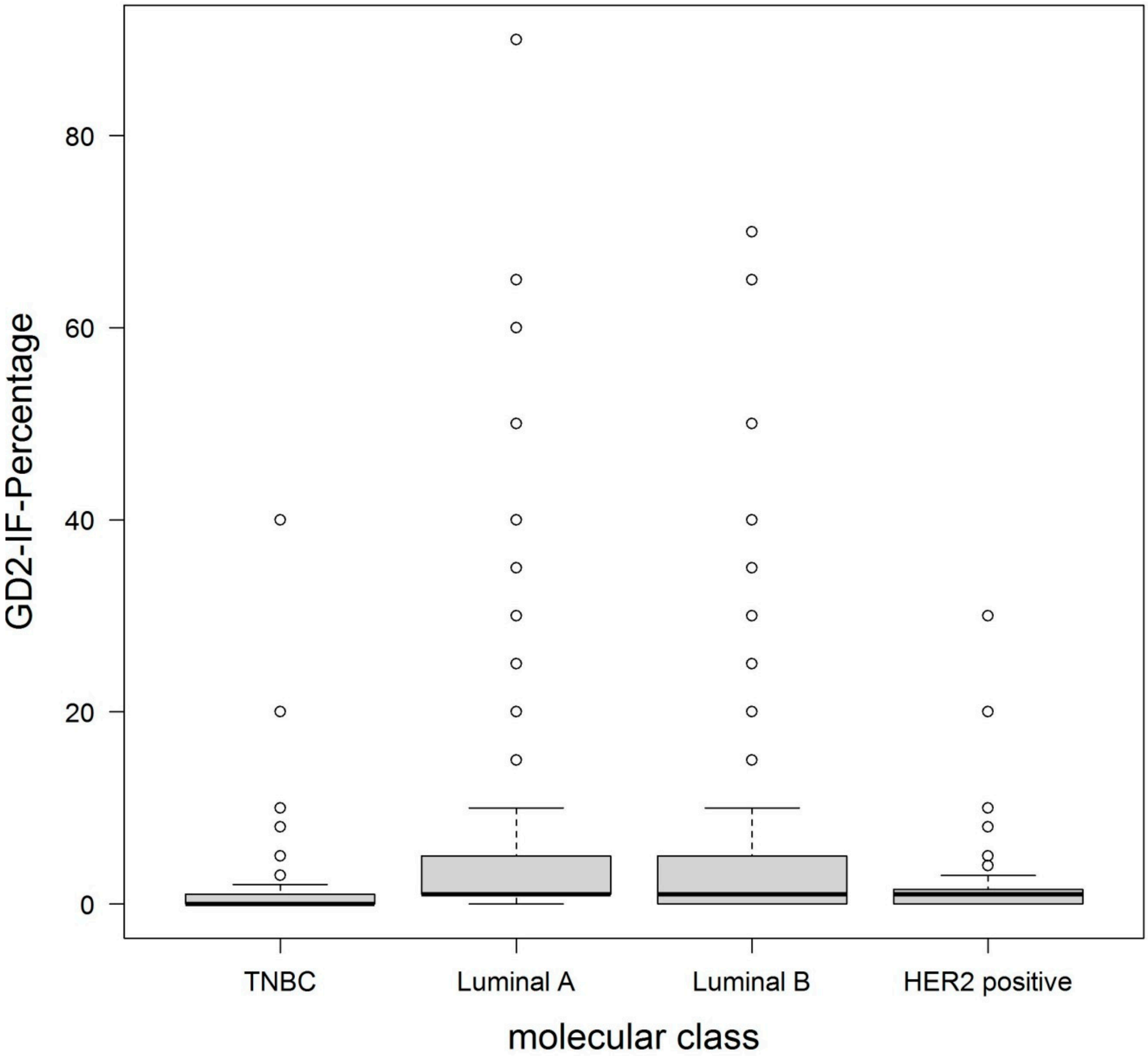
GD2-IF-Percentage
0
20
40
60
80
TNBC
Luminal A
Luminal B
HER2 positive
molecular class

Figure S4. Kaplan–Meier curves for GD2 immunohistochemistry (binary) for disease-free survival (DFS) for *(a)* triple-negative breast cancer patients, *(b)* luminal A breast cancer patients, *(c)* luminal B breast cancer patients, and *(d)* HER2-positive breast cancer patients.

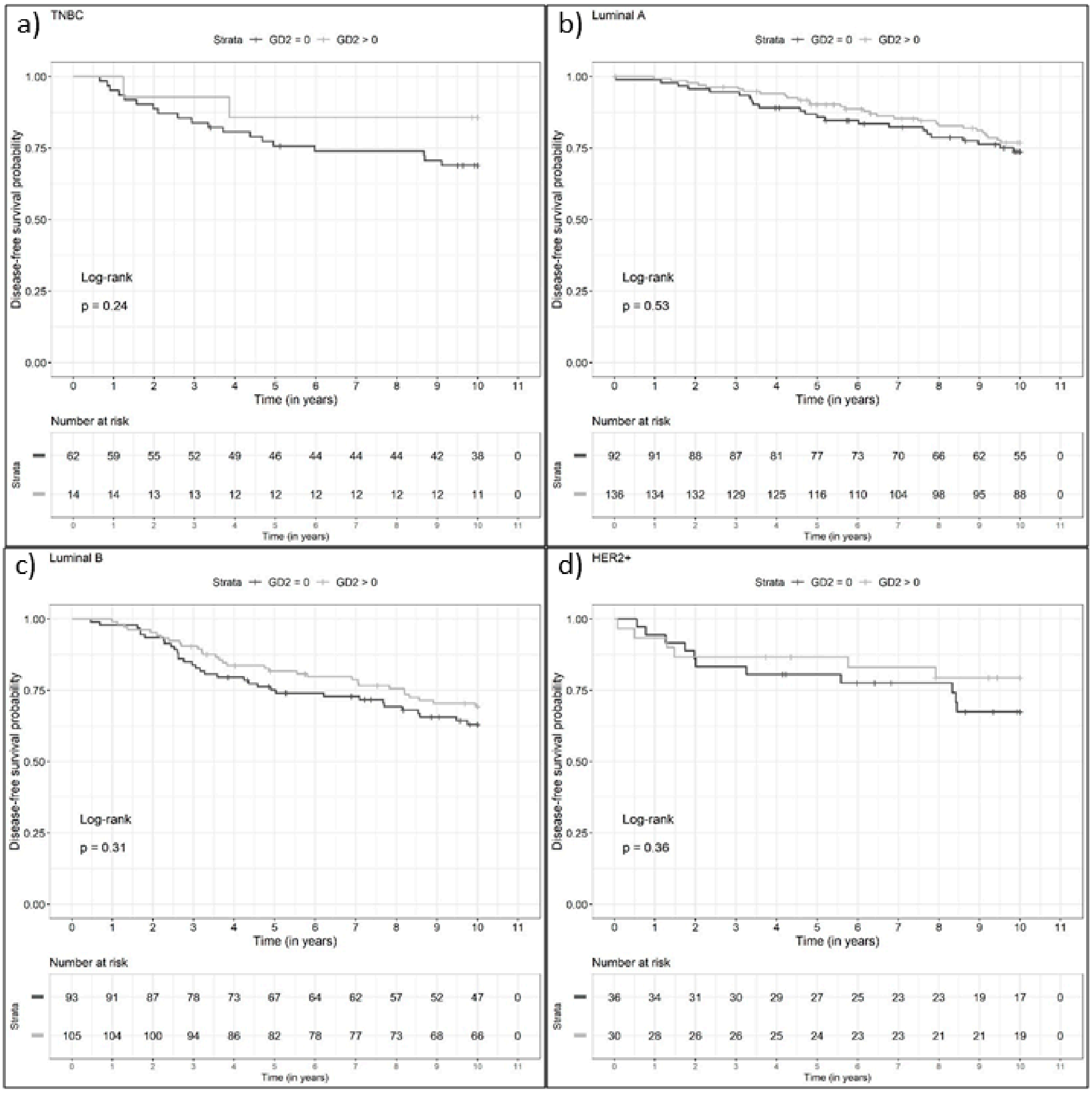

Figure S5. Kaplan–Meier curves comparing overall survival (OS) between patients with GD2-positive and GD2-negative tumors among *(a)* triple-negative breast cancer patients, *(b)* luminal A breast cancer patients, *(c)* luminal B breast cancer patients, and *(d)* HER2-positive breast cancer patients.

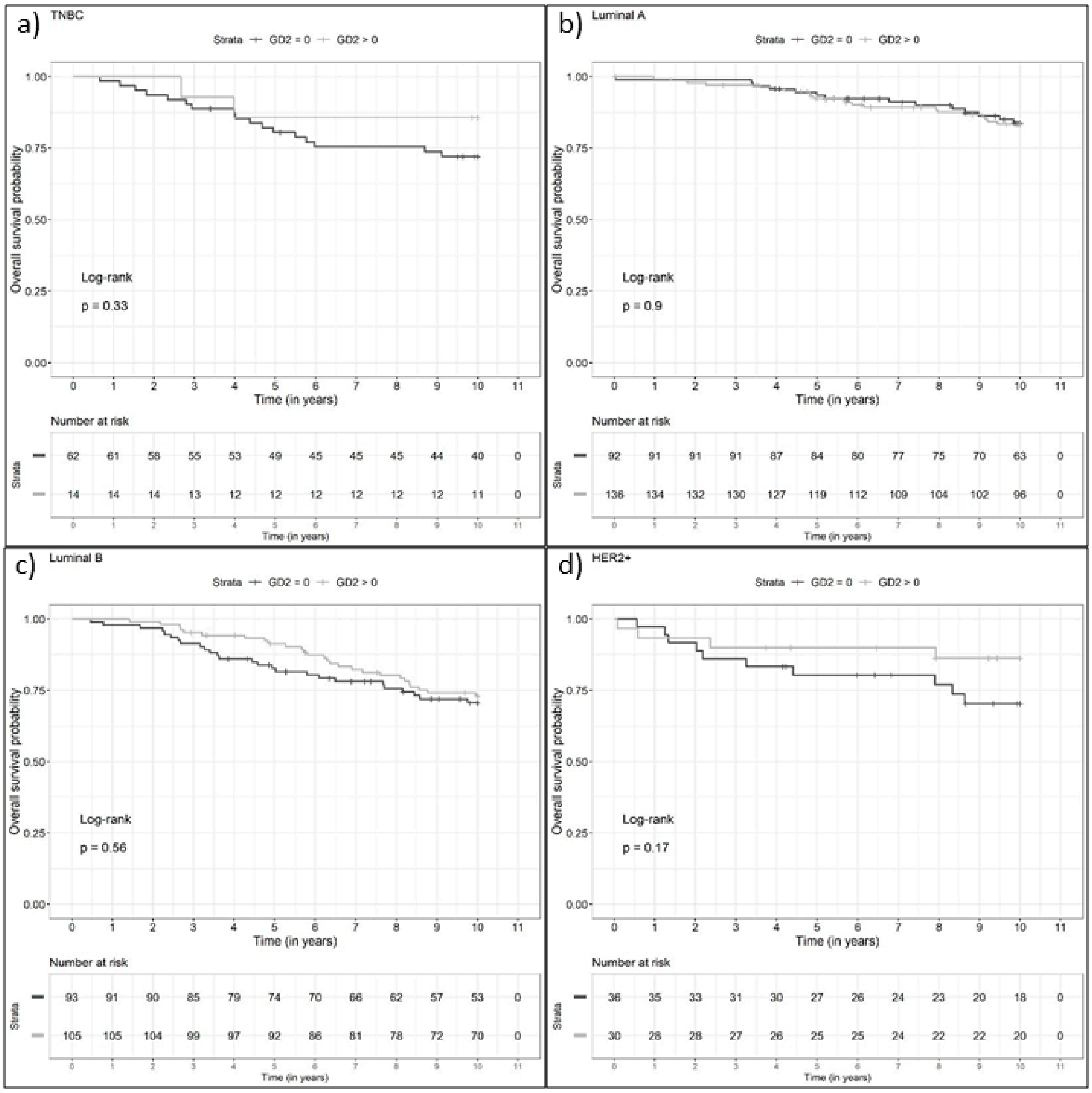

Figure S6. GD2 immunohistochemistry (IHC) in non-neoplastic breast epithelium (original magnification × 200 in 1 and × 400 in 2). The moderate to strong GD2 positivity in a few noncancerous breast epithelial cells should be noted.

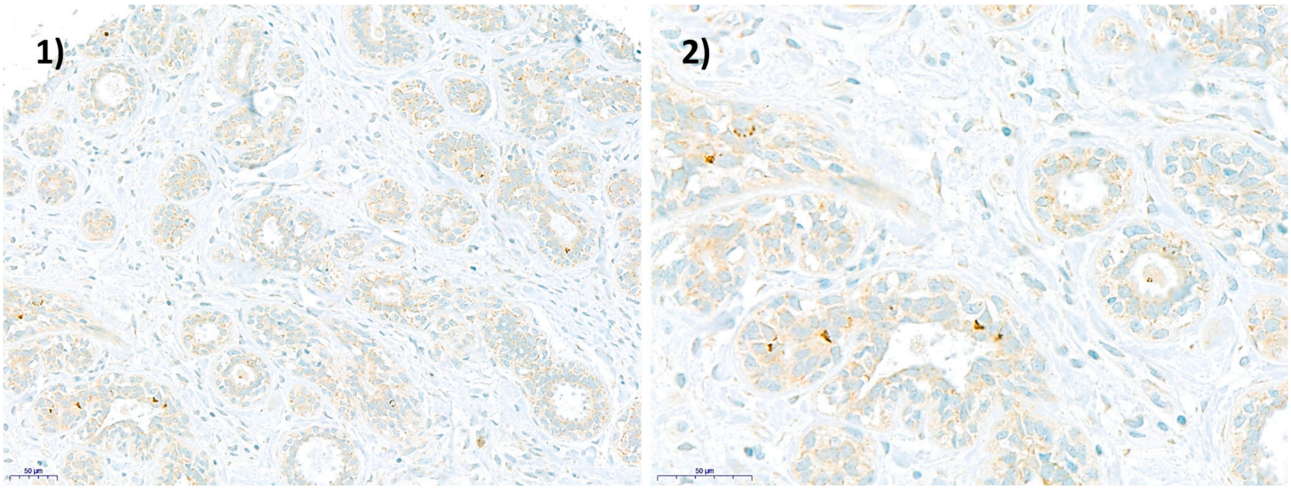

Figure S7. GD2 immunohistochemistry (IHC) of varying breast cancer cell lines. 1) and 2) shows breast cancer cell lines with GD2 positivity: 1) MCF7, and 2) HTB-133 (insets show higher magnification). 3) HTB-132 presented with only weak to intermediate GD2 staining intensity in <0.1% of tumor cells (arrow). 4) HTB-26 did not show any GD2 positivity (original magnification each × 400, insets each × 1500).

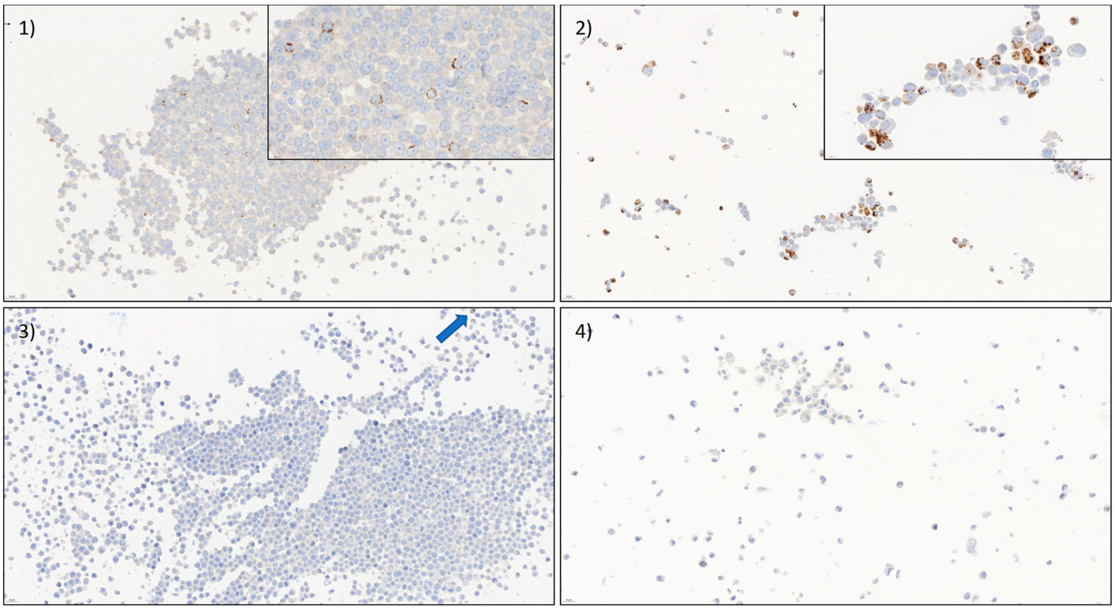

Supplement: Supplementary file 1 [file cancers-13-05577-s001.zip › cancers-1267818-supplementary.pdf]
